# Supplementary material for: Applying machine-learning and deep-learning to predict depression from brain MRI and identify depression-related brain biology
Source: Transl Psychiatry. 2026 Feb 25;16:171. doi: 10.1038/s41398-026-03889-8 (PMC13039452; doi:10.1038/s41398-026-03889-8)
Supplement: Supplementary file 1 — Supplementary Materials [file 41398_2026_3889_MOESM1_ESM.pdf]

Supplementary Information

of

Applying Machine-Learning and Deep-Learning to Predict Depression from Brain MRI and Identify Depression-Related Brain Biology

Jiayue-Clara Jiang (PhD), Camille Brianceau (MSc), Elise Delzant (PhD), Romain Colle (MD,PhD), Hugo Bottemanne (MD,PhD), Emmanuelle Corruble (MD,PhD), Naomi R Wray (PhD), Olivier Colliot (PhD), Sonia Shah (PhD), Baptiste Couvy-Duchesne (PhD)

Supplementary Figures

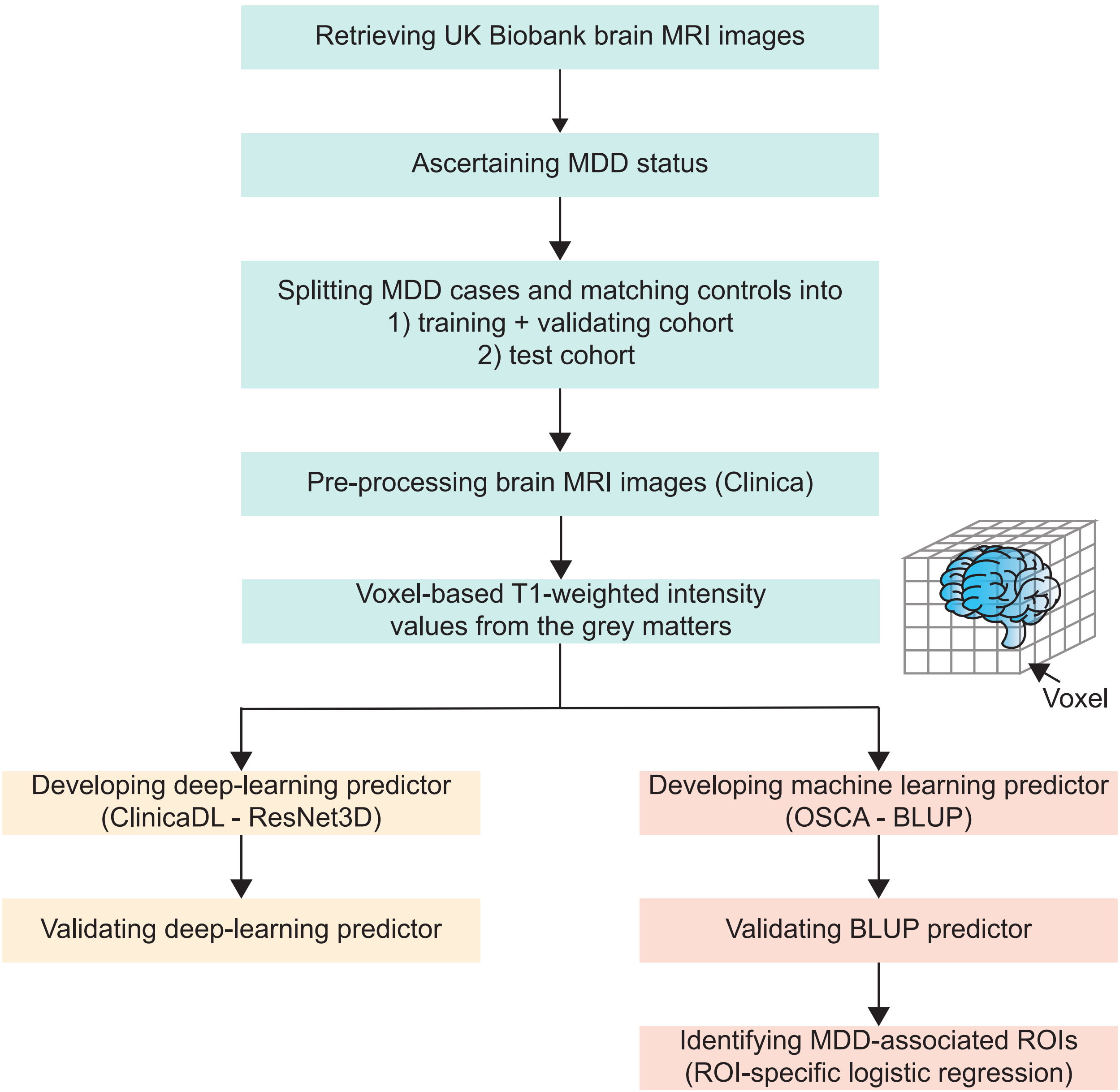

Supplementary Figure 1. Overview of study design.

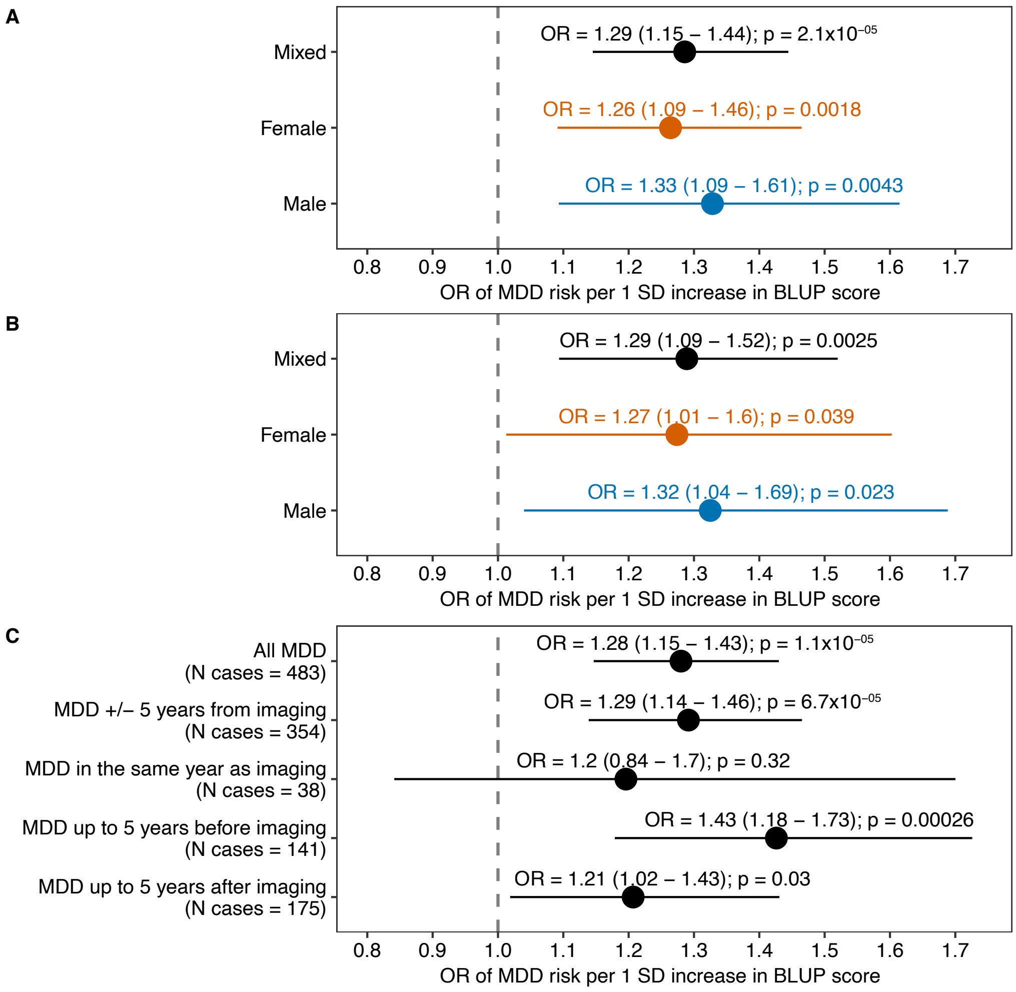

Supplementary Figure 2. Sensitivity analyses of the association between the BLUP predictor and MDD risks. Logistic regression was performed to investigate the association between the BLUP predictor and MDD risks, while adjusting for age at baseline, age at imaging, sex (except in sex-stratified analyses), genetic ancestry, smoking status at baseline, and BMI at baseline. (A) Logistic regression was performed while additionally adjusting for MRI imaging covariates, namely mean rfMRI head motion, scanner lateral (X) brain position, scanner transverse (Y) brain position, scanner longitudinal (Z) brain position, intensity scaling for T1, volume of EstimatedTotalIntraCranial (whole brain), inverted signal-to-noise ratio in T1, and inverted contrast-to-noise ratio in T1. (B) Logistic regression was performed while additionally adjusting for antidepressant use. (C) Logistic regression was performed where MDD cases with a recorded MDD episode more than 5 years prior to or after MRI imaging were excluded from analysis. Logistic regression was adjusted for age at baseline, age at imaging, sex, genetic ancestry, smoking status at baseline, and BMI at baseline. OR represents the change in MDD risks per 1-SD increase in the BLUP score. The grey line represents no association (OR=1).

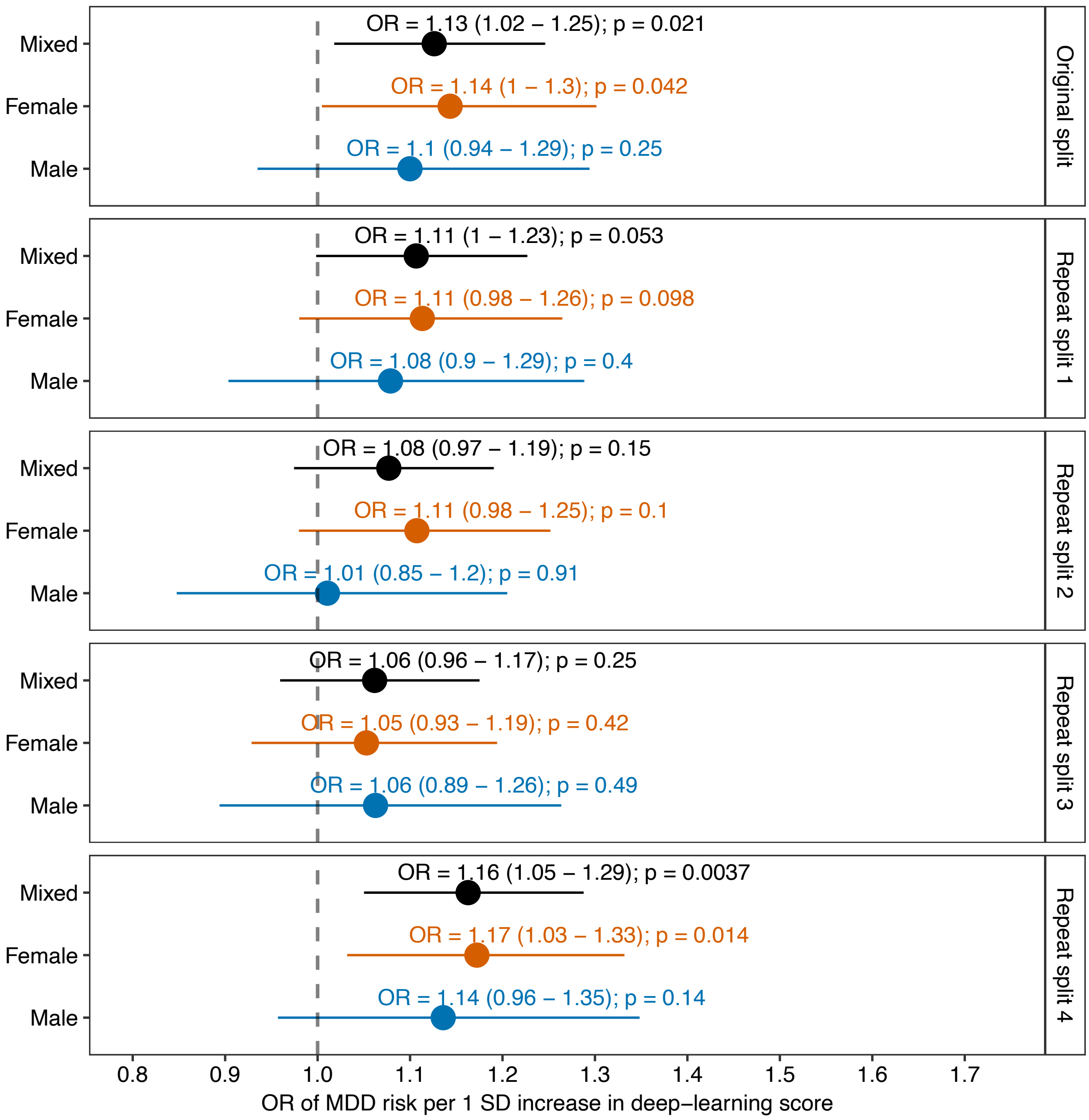

Supplementary Figure 3. Association between MDD risks and deep-learning predictors developed in the repeated splits of the training cohort. Logistic regression was performed to investigate the association between the deep-learning predictors and MDD risks. The logistic regression model included age at baseline, age at imaging, sex (except in sex-stratified analyses), genetic ancestry, smoking status at baseline, and BMI at baseline as covariates. OR represents the change in MDD risks per 1-SD increase in the deep-learning score. The grey line represents no association (OR=1).

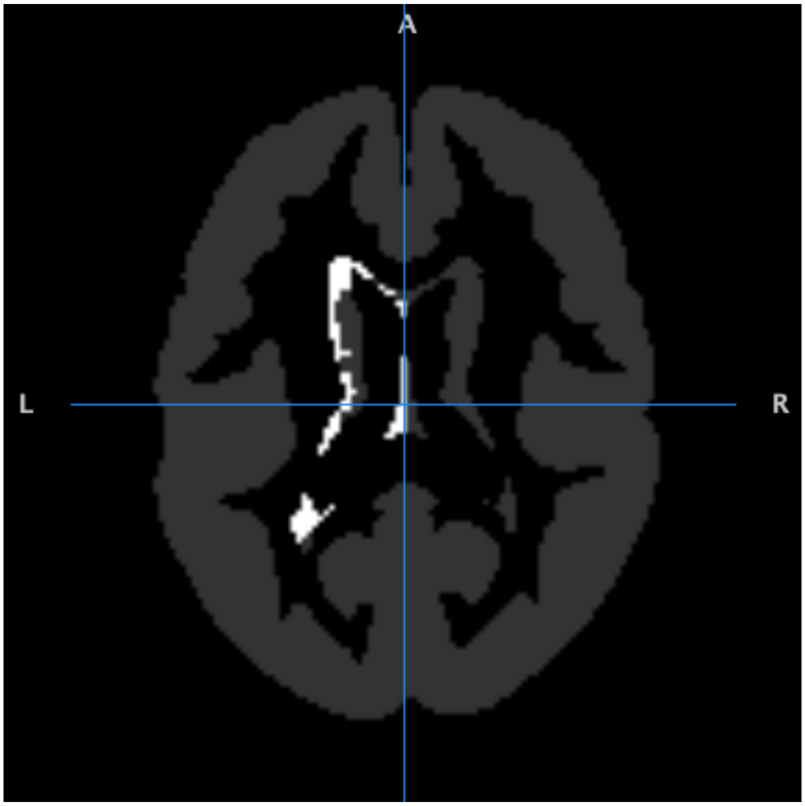

Supplementary Figure 4. Voxels mapping to the left cerebral white matter. Black indicates voxels that were not annotated with a grey matter region of interest. Grey indicates voxels that were annotated with a region of interest. White indicates voxels that map to the left cerebral white matter.

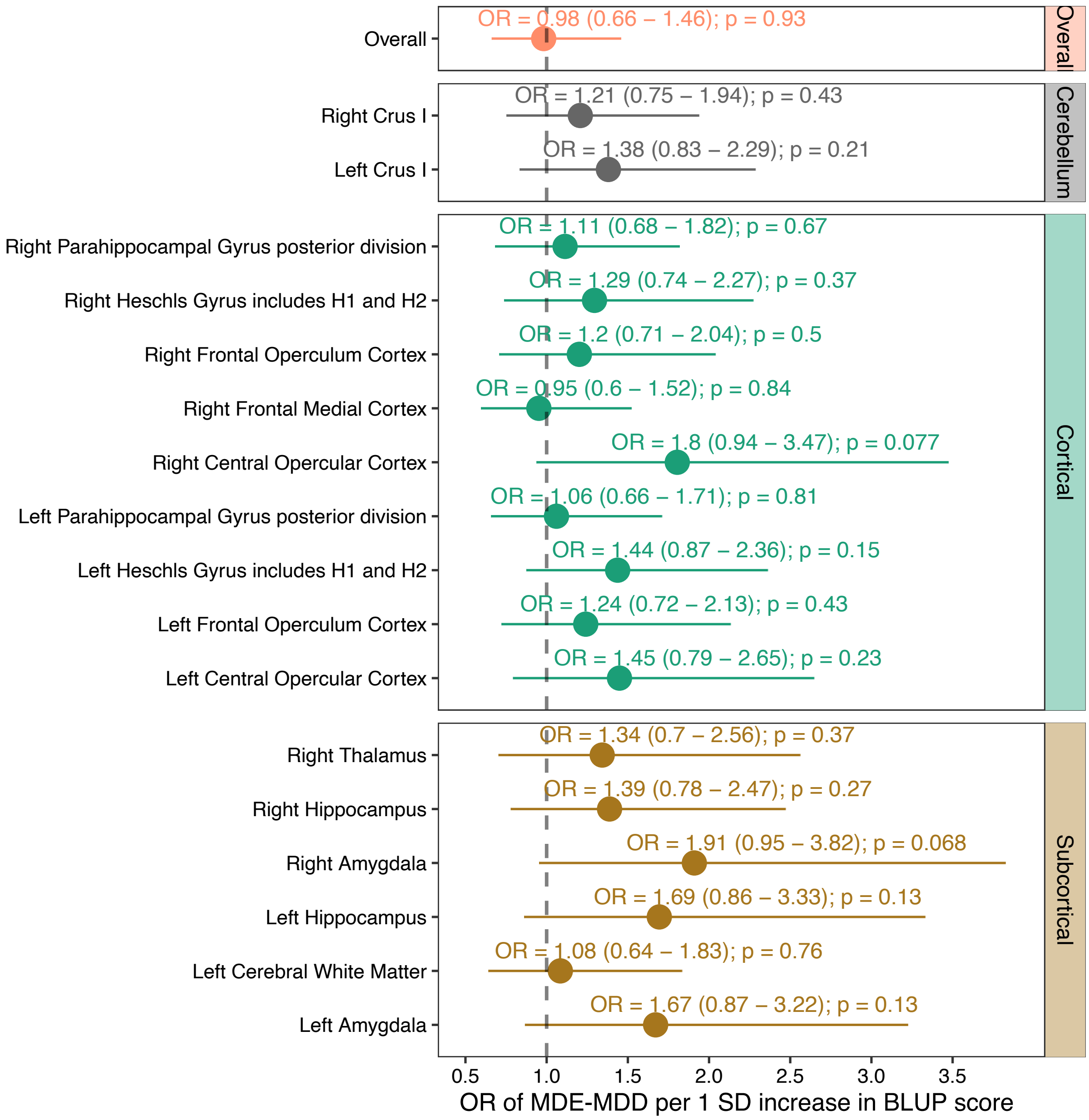

Supplementary Figure 5. Association between MDE-MDD status and the overall and ROI-specific BLUP predictors in the DEP-ARREST CLIN replication cohort. Logistic regression was performed to investigate the association between each BLUP predictor and MDE-MDD status. The logistic regression model included age, sex, BMI, smoking status and ICV as covariates. OR represents the change in MDE-MDD per 1-SD increase in the BLUP score. The grey line represents no association (OR=1).

Supplementary Tables

Supplementary Table 1. UK Biobank MDD ascertainment criteria (BD, bipolar disorder; SCZ, schizophrenia)

Disease	Criteria	Data field	Data field description	Codes
BD cases	Inclusion	41270	ICD-10	F300, F301, F302, F308, F309, F310, F311, F312, F313, F314, F315, F316, F317, F318, F319
		41271	ICD-9	2960, 2961, 2969
SCZ cases	Inclusion	41270	ICD-10	F200, F201, F202, F203, F204, F205, F206, F208, F209, F21, F220, F228, F229, F230, F231, F232, F233, F238, F239, F24, F250, F251, F252, F258, F259, F28, F29
		41271	ICD-9	2953, 2959
MDD cases	Inclusion	41270	ICD-10	F320, F321, F322, F323, F328, F329, F330, F331, F332, F333, F334, F338, F339, F340, F341, F348, F349, F380, F381, F388, F39
		41271	ICD-9	3119, 2962
	Exclusion	BD cases		
		SCZ cases		
		41270	ICD-10	F448
		20002	Non-cancer illness code, self-reported	1289
		20003	Treatment/ medication code	Antipsychotics: 1141202024, 1141153490, 1141195974, 1140867078, 1140867494, 1141171566, 2038459704, 1140872064, 1140879658, 1140867342, 1140867420, 1140882320, 1140872216, 1140910358, 1141200458, 1141172838, 1140867306, 1140867180, 1140872200, 1140867210, 1140867398, 1140882098, 1140867184, 1140867168, 1140863416, 1140909802, 1140867498, 1140867490, 1140910976, 1140867118, 1140867456, 1140928916, 1140872268, 1140867134, 1140867208, 1140867218, 1140867572, 1140879674, 1140909804, 1140867504, 1140868170, 1140879746, 1141152848, 1141177762, 1140867444, 1140867092, 1141152860, 1140872198, 1140867244, 1140868172, 1140867304, 1140872072, 1140879750, 1140868120, 1140872214, 1141201792, 1140882100, 1141167976
Screened controls	Exclusion	BD cases		
		MDD cases		
		SCZ cases		
		41270	ICD-10	F21, F29, F419, F410, F411, F418, F449, F444, F446, F440, F441, F448, F681, F688, F409, F400, F401, F402, F408, F341, F488, F489, F99, F601, F604, F607, F609
		41271	ICD-9	3000, 3001, 3002, 3004, 3005, 3009, 3012, 3015, 3016, 3019
		20002	Non-cancer illness code, self-reported	1286, 1287, 1288, 1289, 1290, 1291, 1531
		20003	Treatment/ medication code	Antipsychotics (see above)<br>Antidepressants: 1140867820, 1140867948, 1140879616, 1140867938, 1140867690, 1141190158, 1141151946, 1140921600, 1140879620, 1141201834, 1140867152, 1140909806, 1140879628, 1140867640, 1141200564, 1141151982, 1140916288, 1141180212, 1140867860, 1140867952, 1140879540, 1140867150, 1140909800, 1140867940, 1140879544, 1140879630, 1140867856, 1140867726, 1140867884, 1140867922, 1140910820, 1140879556, 1141152732, 1140867920, 1140882244, 1140867852, 1140867818, 1141174756, 1140867916, 1140867888, 1140867850, 1140867624, 1140867876, 1141151978, 1140882236, 1140867878, 1201, 1140882312, 1140867758, 1140867712, 1140867914, 1140867944, 1140879634, 1140867756, 1140867934, 1140867960, 1140916282, 1141200570, 1141152736

Supplementary Table 2. Demographic characteristics of the training and test cohorts

		Training cohort		Test cohort	
		MDD	Control	MDD	Control
Total N		987	3934	483	1939
Female (%)		620 (62.8)	2498 (63.5)	314 (65.0)	1218 (62.8)
Age at baseline in years, mean [SD]		54.6 [7.6]	54.5 [7.4]	54.4 [7.8]	54.4 [7.4]
Age at imaging in years, mean [SD]		63.1 [7.7]	63.4 [7.4]	62.9 [7.9]	63.2 [7.4]
Ever smokers, N (%)		466 (47.2)	1844 (46.9)	225 (46.6)	905 (46.7)
BMI at baseline, mean [SD]		28.0 [5.2]	27.9 [5.0]	27.9 [5.0]	28.0 [5.3]
Genetic ancestry, N (%)	European	939 (95.1)	3765 (95.7)	460 (95.2)	1846 (95.2)
	African	5 (0.5)	12 (0.3)	2 (0.4)	10 (0.5)
	East Asian	1 (0.1)	3 (0.1)	1 (0.2)	5 (0.3)
	South Asian	8 (0.8)	32 (0.8)	5 (1.0)	21 (1.1)
	Unclassified	34 (3.4)	122 (3.1)	15 (3.1)	57 (2.9)
Age at MDD diagnosis in years [SD]		61.6 [9.4]	-	61.2 [9.7]	-
